# Social Withdrawal in Preschool Age: A Clinical Case in Intensive Psychoanalytic Psychotherapy

**DOI:** 10.3390/bs13050354

**Published:** 2023-04-24

**Authors:** Fiorenzo Ranieri, Yura Loscalzo

**Affiliations:** 1 Italian National Health Service, Department of Mental Health, UFSMIA Arezzo, 52100 Arezzo, Italy; 2 Department of Health Sciences, School of Psychology, University of Florence, 50135 Florence, Italy

**Keywords:** children psychotherapy, Hikikomori, psychic retreat, social withdrawal, psychoanalysis

## Abstract

In this work, we suggest that children’s social withdrawal might be a precursor of Hikikomori, a phenomenon observed among adolescents and young adults. Hence, psychotherapy interventions with preschool children showing signs of social withdrawal might play a critical role in Hikikomori prevention. This paper presents the case of a five-year-old child treated with intensive psychoanalytic psychotherapy who began therapy due to his refusing to go to school and exhibiting isolating behavior from other children. Among other symptoms were regression, emotional tension, nightmares, and nocturnal and diurnal enuresis. Moreover, the relationship in the family was difficult, both between the parents and between the parent and the child. The intensive psychoanalytic treatment involved three weekly sessions for about a year, followed by six months with one weekly session. Besides illustrating the therapeutic process through clinical vignettes taken from the sessions, this paper also provides clues on how early social withdrawal can contribute to the construction of internal personality organizations that lead to social withdrawal up to self-reclusion (or Hikikomori).

## 1. Introduction

Hikikomori is a phenomenon that, in recent years, has been shown to affect children. It is a particular form of social withdrawal observed for the first time in Japan and exhaustively described by the psychiatrist Saitō [1] as a condition characterized by voluntary self-reclusion in one’s own home and typical of adolescents and young adults who did not show any apparent signs of psychological distress or diagnosed mental illness. Even if initially studied in Japan, there is increasing evidence of its spread in Western countries [2,3], including Italy [4,5]. Therefore, Loscalzo et al. [6] designed an instrument for screening Hikikomori risk in both Eastern and Western countries, with critical implications for the early individuation of Hikikomori risk since adolescence.

However, we suggest that detecting (and treating) the precursors of Hikikomori before adolescence, namely in pre-school children, is vital as the scientific literature showed several cases of extreme social withdrawal in very young children [7,8]. Moreover, through the analysis of the clinical narratives of young Hikikomori, it is possible to identify the role played by the child’s first relationship with the caregiver, which might be repaired thanks to psychotherapy, thus preventing the development of Hikikomori or other forms of severe social withdrawal (e.g., school refusal). More specifically, about the type of mother-child relationship associated with Hikikomori, Krieg and Dickie [9] highlighted a symbiotic relationship between the mother and the child while the father is peripheric. The psychoanalyst Takeo Doi [10] summarizes this type of bond between the mother and the toddler using the Japanese concept of “*Amae*”. This term designs the desire for an exclusive relationship or a passive desire to be loved unconditionally. There are, however, different interpretations of *Amae*’s role in the arising of extreme social withdrawal. For Hairston [11], the *Amae* of the Hikikomori is an infantile way of remaining tied to the mother by refusing growth and the encounter with the real/external world. For Bowker [12], the Hikikomori choice to be self-reclusive is an unconscious request to receive unconditional love from the primary figures who have been missed in the first months of life. In line with this, Mahler et al. [13] presented the importance of the individuation and separation process—after the first phase of symbiosis with the mother—for the child’s psychological development. More specifically, individuation refers to intrapsychic autonomy (e.g., perception, memory, thought process), while separation concerns the differentiation and autonomy from the mother. Disruption of these two evolutive paths might lead to severe psychopathologies, including psychosis [13]. Similarly, Neumann [14]—using a Jungian archetypical approach—suggests that the newborn, in the first stage of psychic development, is connected to the Great Mother in a state of participation mystique (i.e., there is no polarity between subject and object). The Ego is not autonomous but manifests itself as a satellite of the Great Mother or as something that belongs to the Great Mother and is directed by her since the child is in a state of symbiosis. The Ego will gradually arise from the unconscious, taking shape as the center of consciousness, through a series of stages until the higher (or solar) state, where the Ego reaches the connection with the paternal archetype. Hence, the Ego struggles against the Great Mother on the road to emancipation. Therefore, as *Amae* refers to a desired symbiosis with the caregiver, the disruption of the individuation and separation process might also be associated with social withdrawal and Hikikomori.

Regarding social withdrawal, it is important to highlight that infants normally experience transitional stages of isolation during their development since it constitutes a form of protection against tensions deriving from environmental stimuli. The child and his/her caregiver use withdrawal as a form of mutual regulation, thus promoting interaction and the child’s development [15,16]. Social withdrawal interferes with development when it is too frequent or excessively prolonged over time [17]. Usually, it occurs when the caregiver’s ability to contain the child’s anxieties is significantly reduced [18], and there is a lack of a holding environment capable of promoting the development of autonomy through satisfactory primary experiences [19]. For Koshiba [20], in these cases, mothers interpret their role instrumentally but not emotionally; they are overprotective figures that inexplicably become absent. Moreover, the father is usually absent. The child’s relationship with the parents is thus characterized by closeness or distance depending on events beyond child’s control. The mother does not foster the emotional and personal growth of the child in the intersubjective space that the caregiver-child couple should share. In sum, the child becomes the custodian of parents’ idealizations and often supports the mother, trying to maintain a close relationship with her. Consequently, there is an interference with his/her developmental goals, including the possibility of building one’s autonomy by moving away from the maternal figure and exploring the world [21].

In the most unfavorable situations, the child’s frustration—due to an altered child-caregiver relationship—creates the conditions for structuring defensive organizations capable of influencing and characterizing personality development. The child withdraws into fantasy as a defense mechanism: he/she builds an imaginary world dominated by the sense of omnipotence and the refusal of dependence on parental figures [22]. This illusionary world helps the child cope with his/her painful emotions. However, this defensive intrapsychic process might constitute the first step towards constructing a pathological organization of personality, which is the basis for what Steiner named “psychic retreats” [23] and which differs from dissociation, consisting of splitting off mental contents from the conscious awareness and potentially taking the extreme form of dissociative identity disorder [24].

The main signs of psychic withdrawal (and possibly later Hikikomori) in young children might be non-spontaneous interaction with peers, avoidance of contact with others—using the family as a refuge—and regressive behaviors that hinder psychic development. Therefore, the psychotherapeutic treatment of children who show early and significant forms of social withdrawal, besides representing a form of therapy, is especially critical to preventing subsequent clinical disorders, including Hikikomori [25]. In fact, as Muris and Ollendick [26] stated in their recent review of Hikikomori literature, there is consistent evidence concerning the role of behavioral inhibition (i.e., an early temperament feature characterized by the tendency to fear and avoid unfamiliar stimuli, including people) in the onset of psychological disorders linked to severe social issues [27,28]. Therefore, they speculated that behavioral inhibition might constitute a risk factor also for extreme social withdrawal, or Hikikomori [26].

This paper presented an 18-month intensive psychoanalytic psychotherapy treatment with a pre-school child presenting early signs of social withdrawal as an illustration of the treatment of a child with potential later onset of psychopathologies linked to social issues (such as social anxiety, school refusal, and Hikikomori) due to the presence of early signs of social withdrawal in pre-school age. The child participated in 3 weekly treatment sessions in the first 12 months. Then, in the last six months, the frequency decreased to one weekly session. Constant contact between the therapist and the mother was maintained during treatment. The child’s father (separated from the mother) participated in one couple and three individual meetings and avoided other contacts with the psychotherapist. The psychotherapist is a qualified psychoanalytic psychotherapist at the Marta Harris Study Center in Florence (Tavistock Model) and works for the National Health Service at the Department of Mental Health, Childhood, and Adolescence Unit. The case material has been anonymized, changing the names and other relevant information which might lead to the recognition of the child and his family. 

## 2. The Clinical Case 

The psychotherapist meets Samuele’s mother, Sara, in an Italian National Health System clinic. The woman explains she came alone because her relationship with Fabio, Samuele’s father, is complicated. The parents do not live together—they separated a few years ago—and their two children, Anita and Samuele (the second born), live with the mother. Samuele and his older sister meet their father twice a week and on alternate weekends. On these occasions, during the meeting between the parents, heated arguments have sometimes led to the call of the police. The father now lives with another woman, while Sara has a stable emotional relationship with a (not cohabitant) woman of her same age. Anita and Samuele know this woman as an “aunt” (instead of as the mother’s partner). 

Sara is worried about many symptoms shown by Samuele, who is five years old at the time of the meeting with Sara. In the last few years, Samuele seems to have regressed and sometimes behaves childishly (e.g., he eats with his hands). The child seems emotionally tense, has nightmares, and grinds his teeth during sleep. Sometimes he wants to sleep with his mother. Moreover, there were frequent episodes of nocturnal and diurnal enuresis. Though, the issue that worries the mother the most is Samuele’s difficulty in going to school. Samuele refuses to go to school (i.e., kindergarten) and—according to teachers’ report—when he attends the school, he stays for many hours on the sidelines.

Following this first interview with the mother—considering the high conflict between the parents—the psychotherapist decides to meet the father and mother separately, and he invites Fabio. He informs the psychotherapist that he has a daughter with his new partner and states that keeping secrets is one of Sara’s traits. According to the father, the children are “educated” to secrecy: in his view, Samuele’s tension is due to the many secrets and unsaid things in the mother’s family. From this interview, the psychotherapist understands that the father frequently questions his children to reveal his ex-partner’s secrets.

The two separate meetings with the father and mother achieved a significant result: both parents consented to the psychotherapist encounter with the child and possible treatment.

### 2.1. The First Session with Samuele

Samuele arrives with his mother on time. He is short, though proportionate and thin. He seems shy, and his mother must encourage him to enter the room. Once inside, the first object he takes from the toy chest (placed on a low table) is an openable transparent box, similar to a casket. The box is empty: Samuele opens it and immediately puts in some pieces of wooden constructions. He then takes other items out of the toy chest and puts them in this little box. The therapist comments that he is putting everything that seems precious to him in the box, and Samuele replies yes, nodding his head.

Samuele appears as a child who needs—rather than playing—to grab precious things and put them aside. The first approach with the room and the therapist is a greedy emptying with an appropriation of the precious objects contained therein [29]. It can be deduced that Samuele feels like an empty container that needs to be filled; however, he enacts a devour that does not satisfy hunger. Hence, we suggest that Samuele is in a condition of deprivation in which he must provide for himself, grabbing everything that seems good. The continuation of the session confirms this hypothesis.

In particular, Samuele takes the glue and spreads it a little on a sheet. He asks for something to be attached, and the psychotherapist replies that he could attach another sheet to the one he spread with glue. Samuele instead takes a bale of hay and tries to stick it without success. He then asks if he should add more glue. The therapist does not answer the question, and, in the meantime, he signals to the mother that she can go away, and Samuele continues to play calmly.

Through the glue game, Samuele seems to communicate his need to bond with the object but does not know how to do it. He cannot establish a relationship between two similar materials (sheet and sheet), so he seeks relationships with different materials (sheet and bale of hay). The psychotherapist feels the need to convey the message that the child can find a safe space in the psychotherapeutic room to communicate his needs with an available adult. A closeness that does not hinder the game but serves the purpose of understanding it. Samuele then feels that he can allow his mother to leave.

The child abandons the glue game and continues to take objects out of the basket and place them in the box with the lid or on the table. Then, when done, he puts cutlery for two people and two plates on a sheet of paper and saucepans. The child’s order in arranging the objects strikes the psychotherapist, and he comments that Samuele seems ready to cook. Samuele realizes that there are some plasticine single-dose packs in the toy box. He tries to open them unsuccessfully; hence, without saying anything, he hands them to the psychotherapist to have them opened. Samuele says to know a child who, while playing with plasticine, really ate it. Then, he adds that he has decided to prepare a soup of white plasticine.

In this initial session, Samuele seems to probe the characteristics of the psychotherapist and whether it is possible to trust him, as the mother took the child to the psychotherapist. The psychotherapist demonstrated that he does not intervene in the games even if the child greedily empties the basket by appropriating the best pieces. Moreover, when asked for help, he is present. With his latest communication (i.e., the comment about the child who played with plasticine and then really ate it), Samuele questions if the psychotherapist can stay in an intermediate area which is like reality, but it is not reality at the same time. It is essential for Samuele to feel that the psychotherapist can help him stay in a shared space in which their reciprocal play abilities overlap: the psychotherapist must be able to understand the limit between play and reality [30]. Without waiting for an answer, the child immediately tests the psychotherapist: if he eats the plasticine soup, he will be too “crazy” or too “child” and therefore unreliable.

While the child and the psychotherapist play with plasticine, Samuele says that his schoolmates make fun of him when he is at school. When this happens, he gets furious, scaring and making all the other children run away. All except a little girl named Giulia. She is his friend and is not afraid of Samuele. There is also another child who does not run away, Ettore. Samuele explains that Giulia is engaged to Ettore. Instead, he is not engaged to Giulia and is just a friend (he repeats this twice).

Therefore, the essential point for Samuele is to believe that the psychotherapist is sane enough not to eat the plasticine soup and strong enough not to get frightened when the child gets angry. This is important for being close to Samuele while complex triadic combinations appear in his mind. Therefore, the possibility of a connection between the child and the therapist appears as a possible event, and Samuele can now attach the two pieces of paper using glue. Projective identification seems to have worked well enough [31]. The psychotherapist and child can meet and establish contact.

**Figure 1:**
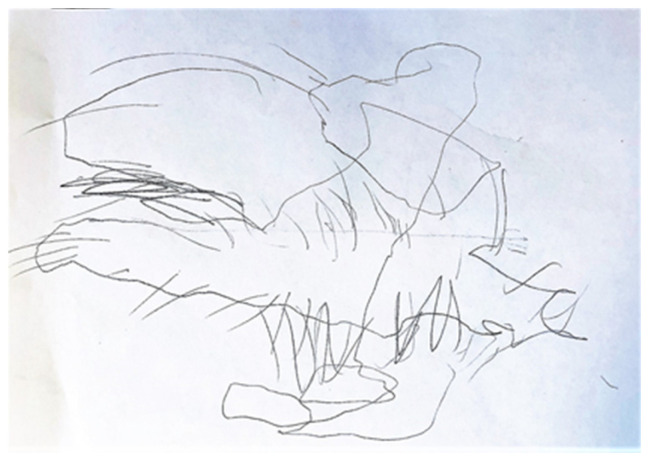
The giant squid drawn by Samuele at the end of his first session.

At the end of the first session, the psychotherapist announces that it is time to leave and invites the child to help him put the toys away. Samuele decides to draw instead. He makes a long scribble on a piece of paper and asks what it could be. Obviously, the psychotherapist does not know. Samuele says it is a giant squid and continues to add pieces and details to his drawing until it almost covers the entire sheet (Figure 1).

Inside Samuele is a giant squid, a monstrous being that perhaps activates itself at the moment of separation. This gigantic entity—which takes up the entire sheet/inner world of the child—must be known, understood, and perhaps remodeled. At the end of the session, the child presents the question on which the therapeutic couple will work.

After this first meetings, the psychotherapist proposes to both parents a three-session treatment per week for approximately one year.

In sum, in the first session, Samuele showed the therapist his core therapeutic themes: he has a separation issue and aggressive tendencies that need to be addressed by the therapy to allow him to recover his developmental path. Samuele must gradually go through his individuation and separation process to find the proper openness to social life outside his symbiosis.

### 2.2. First Stage: Cannibalism towards the Objects

In the first months of therapy, Samuele and the psychotherapist encounter profound anxieties. In this phase, Samuele’s attempts to manage his anxieties are characterized by low-level defenses, the only ones available to him. In particular, the psychotherapist is confronted with the “cannibalistic” dimension of the child, where eating or being eaten appears to be a real possibility. The third session is an example of this aspect.

Samuele gives the psychotherapist two pieces of plasticine for him to open. The child sniffs the pink piece and says “this tastes like strawberry”. He then sniffs the black piece and says “this smells like poop”. Both times, after his verbalization, he also lets the therapist smell the plasticine. Samuele then quickly works the pink plasticine and makes it into a ball. Then he rolls out the black plasticine and wraps the pink ball. Finally, he encases the black plasticine (which itself contains the pink plasticine) with a layer of orange plasticine. The psychotherapist comments that each piece disappears into the other and still exists but can no longer be seen. Eventually, the orange is large enough to envelop the black completely. At this point, Samuele says, “Do you know? There are some children at school who always make fun of me”. He adds: “These children want to eat me”. This cannibalistic image and comment strike the psychotherapist, who says, “Perhaps you would like to make them disappear in a layer of plasticine!” Samuele nods, convinced. The psychotherapist, thinking of all the times Samuele asks not to go to school, adds “or maybe sometimes you would like to disappear!” Samuele nods with even greater conviction.

Thus, the (good) pink plasticine is enveloped in the (stinky) black plasticine, which in turn is wrapped in the orange plasticine. The encounter between good and bad generates dramatic persecutory anxieties that require drastic defenses. The psychotherapist tries to transform the anxieties into thinkable content, carrying out a function of containment and transformation, carefully communicating his understanding to Samuele so the child can understand it [32].

The psychotherapist also becomes the object of cannibalistic attacks in the same session. After a phase in which two groups of animals fight and devour each other, Samuele transforms the clash between the animals into a conflict between him and the therapist. He says they have to fight with a fork and knife. In a humorous tone, the therapist comments, “So, if you hit me, you eat a piece?” Samuele nods in amusement. The fight begins. Samuele hits the therapist on the arm and says that he took a piece of meat and ate it. Then, he pokes his belly and again says he took a piece of his flesh. He seems proud of his victories.

In Samuele’s games, there are commonly two opposing fields, with roughly equal forces and an intermediate space that divides them. Two groups of animals, soldiers, or human beings face each other and eventually clash until one of the parties succumbs, sometimes with scenes of cannibalism. Even the human figures are cooked on the stove and eaten.

In summary, in this first stage of the therapy, Samuele shows his low-level defenses for managing his anxieties, mainly related to oral/cannibalistic themes. 

### 2.3. Second Stage: The Equivocal Dimension of Objects 

In a subsequent stage, the contrast between parts that collide up to the total cannibalistic introjection of the other becomes even more complex with the introduction of the equivocal dimension of the objects. What looks good can turn out to be bad, and what is bad can masquerade as good: orientating in the game is impossible.

The 19th session is an example of this aspect. Samuele suggests making a labyrinth. The child then begins to move all the chairs to shape a path that leads to an empty space. The path will be littered with mines represented by plastic (Legos) constructions. Samuele begins to take Legos from the basket and throw them randomly on the floor. The child comments that there are many mines in the basket, and the therapist thinks that his mind is full of destructive objects. The floor soon fills up, making the ride bumpy. At this point, Samuele takes the wooden buildings and explains that these are not mines but weapon reloads, a system for recharging and having more energy. The wooden pieces are good versus the lousy plastic constructions. He starts throwing the pieces of wood. Of a yellow piece of wood, Samuele says “this is a mine” and puts it on the ground. The therapist comments that this makes everything very difficult; if a piece of wood is a mine, one will never understand it. He also adds that bad things masquerading as good things are confusing and dangerous. Samuele insists that the yellow wood is a mine. Furthermore, a piece of Lego is now transformed into a good item. The therapist protests and Samuele stops masking the pieces but starts another game. He takes a good piece (wood) and surrounds it with bad pieces (mine). In this way, it is impossible to be able to take the good things that are in the object. The therapist says that perhaps the child is talking about how difficult it is to get in touch with good, nourishing things without getting hurt. Samuele begins to rub the pieces of wood one at a time between his hands to recover all the energy. He repeats this operation four or five times, then, once he has “filled up”, he picks up one of the mines. The therapist says that in this way, the mine will explode, and indeed the mine explodes, but it is a small explosion that Samuele can handle after he has charged himself with energy that protects him. Thus, several mines explode without damage. Each exploded mine, like each piece of wood emptied of weapon reloads, is thrown into the basket. In the end, the floor has fewer obstacles. At this point, the child and therapist can transition more easily. Samuele invites the therapist to walk the route with him to the exit. At the end of the labyrinth, there is a tiny corner where the child and therapist hole up.

The therapist tries to understand why there are so many “false” objects in Samuele’s mind. These suspicious objects make it difficult to get good nourishment and recognize people, emotions, and relationships for what they are, helping the child to grow and enrich his mind. Melanie Klein argues that one of the most important defenses against envy is confusion. Due to projective and introjective identification, not distinguishing between the goodness or badness of a substitute for the original figure counterbalances the persecution and the sense of guilt for damaging and attacking the primary object with envy [29]. However, we suggest this is not Samuel’s condition. The child accurately describes his objects; hence, it is possible to argue that he knows them well. They are equivocal, masked, multi-layered objects. Though, this relationship with objects that have the characteristic of representing themselves in changing forms undermines the possibility of trusting them. Samuele only trusts dead objects, personally killed, which he devours cannibalistically and do not satisfy him. Moreover, greed is based on a form of introjection carried out with anger. The violence of oral incorporation, which involves the act of biting, leads in fantasy to the destruction of the object: the hunger that gives rise to more hunger is called greed [29]. Instead, the child prepares the food by himself, with the omnipotent fantasy of being able to feed himself without needing others [33].

Family relationships do not help the child regain trust. There are weekly transfers from mother to father, and each transfer implies a transformation of the object. The mother, to whom Samuele is very attached, is attacked and denigrated when the child is with her father, and vice versa. In this way, the objects that give energy are transformed into dangerous mines, and the familiar reality is represented as a minefield. The many secrets of the mother do not help; moreover, there are intrusion attempts by the father, who tries to steal information about the ex-companion through the children.

Therefore, in this second stage of the therapy, Samuele highlighted, through his games, the presence of “false” objects in his psyche, introducing the equivocal dimensions of the objects.

### 2.4. Third Stage: The Rage towards the Objects 

Psychotherapy turns into the possibility of finding a salvific path that allows Samuele to find a quiet corner, an area free from conflicts. Once Samuele gets rid of the masks that cover him, it appears a deep anger toward the object that risks destroying its possibility of being a resource. His attacks on the object/breast appear evident at times.

In one session, particularly in the 26th session, Samuele models a watermelon with plasticine. He then starts to mark the surface with cuts made with a toy knife. Finally, he cuts the watermelon in half and into slices. The nutritious watermelon is transformed a few minutes later into a character that the therapist must interpret and who collides with a hamburger, played by Samuele. The nourishing breast has become an enemy. 

In the following session (the 27th session), Samuele begins to manipulate the green plasticine and asks if it is possible to make a giant watermelon like a real one. After having fiddled with plasticine for a while, Samuele takes a toy soldier with a rifle that mounts a bayonet. The toy soldier hits the plasticine/watermelon piece with the bayonet, going deep. The therapist feels the scene represents attacks on the breast by a sadistic penis. After three or four strokes, the therapist tells Samuele that he finds it strange how he treats the watermelon. The watermelon has some good stuff; it is good to eat, but he is hitting it by ruining it. Samuele does not seem to listen; he pulls the toy soldier away, takes a piece of construction, and places a cow (with evident udders) on it. The toy soldier approaches the cow and shoots it. He then takes some other animals, especially a lioness and an antelope with big horns, and places them next to the dead cow. It seems that the toy soldier is about to shoot them too, but at this point, Samuele says, with a slightly frightened expression, “Now the lion is coming!” The soldier moves back and hides behind the box’s edge. The therapist tries to tell the child that he seems to be afraid of a reaction to what he did: he hit the watermelon first and then the cow, and now he expects a revenge.

As the therapy progressed, Samuele allowed himself to show his deep anger towards the (mother) object, with important implications for his individuation and separation process.

### 2.5. The Parents’ Reactions to the Therapy 

It is important to highlight that, as psychotherapy proceeds, the games in session become progressively less bloody, and some symptoms regress after a few months. The enuresis disappears, and the child gradually returns to kindergarten. After several months, the therapist has the impression that the child begins to trust the therapy.

To fuel this positive therapeutic process, the psychotherapist has contact with both parents, believing that it is essential to associate collaborative work with the parents (or the family) with the individual psychotherapeutic treatment addressed to the child [34]. After several individual meetings with the mother and a meeting with the father, he manages to organize a meeting for the couple. The therapist explains how important it is for Samuele to have parents who allow him a less problematic transition from one to the other. Unfortunately, the meeting does not give the desired result: a week after the meeting, Samuele’s father asks for an individual session in which he communicates that he does not want to meet her ex-partner again. After that, despite numerous invitations, Samuele’s father will no longer encounter the psychotherapist. Even the mother seems upset by the failure of the mediation meeting. The woman does not directly express her disappointment, but on several occasions, she declares that she feels tired from the rhythm of three weekly sessions for Samuele. Furthermore, she consults another psychologist and then a psychometrician, introducing Samuele to a rehabilitation program, which is helpful for Samuele’s difficulties in writing.

Samuele seems to feel these attacks on therapy and devises ways to reassure himself that he will not separate from his therapist. At the end of each session, he pretends to write a word on an object or piece of plasticine, which he then places in the transparent box. Returning in the following session, he restarts from the mysterious word left as a magical guarantee for a new meeting.

Therefore, Samuele shows progressively higher independence from the mother and establishes a relationship with the stable object represented by the therapist, indicating a further important step towards his individuation and separation process. 

### 2.6. The Last Stages of the Therapy

Samuele is always very active and engages the therapist in many ways. Inspired by a popular video game (i.e., Fortnite), Samuele unleashes himself in battles that see the therapist as a co-protagonist. His need to control things is evident as he often feels controlled by the events. The therapist’s countertransference is to feel as fragile and exposed as the little patient. Psychotherapy goes on thanks to a balance that can be destroyed at any moment. However, there is deep solidarity when the therapeutic couple is in the room. Samuele sometimes experiences extreme emotions, and the therapist finds it spontaneous to stop him, i.e., to help him get out of the game. These are moments when Samuele enacts deeply destructive characters such as a vampire or a warrior who has no mercy for anyone. The child then seems shocked by the destructiveness he shows in play, annihilated by what he sees of himself. Containing him and helping him become a child again gives him a sense of relief. Progressively, Samuele can imagine a reparative process, namely, an alternative to the pure evacuation determined by destructive attacks, as exemplified by the 56th session. Samuele is preparing a battle with the toys in grand style, but the session is now over. He then begins to put the objects in the basket with a lot of energy and speed and asks to set apart two ships assembled with the constructions. He leaves them last, then throws them into the basket with great force, to the point that the constructions break apart. He says, “After all, they were ugly!”. The therapist comments that, instead, they did not look bad. Samuele replies that they would have made more beautiful ones next time.

In the final stage of the psychotherapy, Samuele reports significant improvements: the child has started primary school, which he attends without problems; enuresis and regressive behaviors disappeared; the initial scholastic difficulties are stemmed and progressively overcome (to the great relief of the mother). However, at this stage, there is a further attack on the therapy by the parents. The lack of mediation between parents and the subsequent self-exclusion of the father from the therapeutic process leads the latter to clear sabotage of psychotherapy. Samuele begins to miss the sessions during the days at his father’s home (without any communication). Attempts to be in touch with the father have no effect like the messages sent to him through the mother. On the other hand, the mother appears to feel a sense of gratitude. The woman renounces using psychotherapy as a weapon against her ex-partner (even if his conduct—harmful to Samuele’s needs—would have allowed it), and she is happy with the changes she perceives in the child.

**Figure 2:**
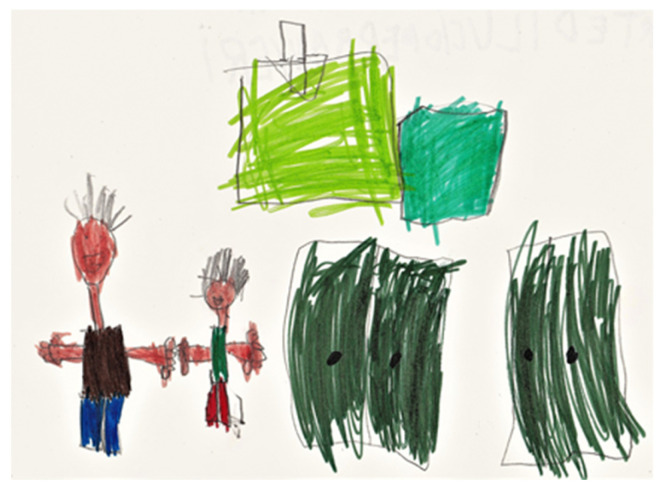
Samuele’s drawing: Samuele, the psychotherapist, and the room’s closets.

Close to the agreed conclusion of the psychotherapy (one year), the therapist offers the mother a further six-month treatment period (one weekly session). In one of the last sessions (the 79th), Samuele arrives with a drawing. On the back is written, “by Samuele to Fiorenzo (the therapist’s name)”. In the drawing (Figure 2), Samuele shows who he is and who the therapist is. He then indicates the two closets in the room, which are closed with the other childrens’ toys and consumable materials, that the child had eagerly desired in the early stages of psychotherapy. Hence, compared to the first drawing, it illustrates the change in the child’s inner world during therapy. There is no longer the giant squid of the first session but an inner space in which the sense of trust in the adult allows Samuele to manage destructive emotions such as greed and intrusiveness. Therefore, the separation and individuation process seems to have been reached by the end of the therapy.

## 3. Discussion

The illustration of the clinical case highlighted how the child tried to defend himself from the network of complex and unclear relationships that surrounded him. The clinical material revealed that Samuele’s inner world, at the beginning of the psychotherapy, was structuring a self-referential system based on omnipotent fantasies. Samuele, in his mind, felt capable of recognizing and avoiding the many equivocal contents he had experienced in his relational world. De Masi [35] argues that the lack, or deficiency, of child-caregiver relational experiences capable of structuring the mind by nourishing the emotional and communicative functions constitute an emotional trauma. This trauma in the primary relationship often drives the child to relational withdrawal and to the construction of pathological structures that could accompany him for the rest of his life. However, social withdrawal experienced as a solution to mental suffering makes the child vulnerable. Withdrawal—preventing the perception of the parents’ absence and the sense of abandonment while creating a state of pleasure—progressively damages contact with emotional and relational reality [36].

Therefore, the psychotherapist had as a first objective the construction of a space—first of all mental—in which the child could feel able not to have to pretend and, hence, rediscover a sense of trust in the adult. In other words, he built a setting capable of instilling trust in Samuele [37]. The treatment was then strengthened in conjunction with Samuele’s ability to entrust himself to the setting, thanks to the continuity of the sessions, the function of containment and mentalization offered by the therapist, and the therapist’s ability to resist the attacks that came in different forms from the more destructive parts that dwelt in the patient’s inner world.

On a reality level, the therapeutic relationship also had to deal with the attacks brought by the parents, who decided to use psychotherapy as a weapon to be used in their endless conflict. In the clinical case presented, the role played by the articulated and conflictual relationships between the parents in constructing the child’s withdrawal behavior is particularly evident. The relationship between family dynamics, intrapsychic processes, and reported symptoms was transparent during treatment. As a child approaches the age of five, primitive Oedipal anxieties tend to give way to triangular battles. Samuele seems to have encountered more significant difficulties than other children in dealing with this period, given the complex interaction between character and experience [38].

However, the construction of shared mental space has made it possible for Samuele to recognize needs, share emotions, and transform contents acted out into contents somehow expressed in the game and, therefore, as thinkable thoughts [39]. A system based on the omnipotent ability to do it alone has been progressively replaced by a relationship that would allow the child to see and be seen [40], emerging from the asphyxiated mental condition that reassures and, at the same time, imprisons.

## 4. Conclusions

This paper presented an intensive psychoanalytic therapy addressed to a preschool child exhibiting various symptoms, especially social withdrawal and school refusal. The therapist conducted the treatment using the Tavistock method [41]. The therapy led to a substantial remission of the symptoms. However, the most critical results concern the (positive) influence on the child’s personality. 

The process related to the personality’s construction (including its pathological organization) begins from the child’s first interactions with his/her family environment [42]. The mental and social functioning of children with a pathological personality organization—if maintained over time—hinder the lines of development and a balanced personality organization, representing a potential precursor of that form of social withdrawal known as Hikikomori [43]. Early forms of retreat lay the foundations for highly structured and steady defense mechanisms and internal object relations that provide the individual with a mental place to remain relatively quiet, protected against the strains of contact with others [44]. This form of mental functioning, shaped by the sense of omnipotence, takes over when reality is felt too frustrating and distressing. Psychotherapy offers the child the opportunity for human contact that welcomes the projections and progressively allows him/her to establish a dependence on a good object. If the psychotherapist demonstrates to be capable of resisting envious and destructive attacks, the child may succeed in attenuating the greed that dries up and makes the acquisition of knowledge impossible. Attempts to place trust in objects—initially experienced as fragile and loaded with ambivalence—gradually increase by replacing the false certainties provided by social withdrawal. The child experiences how authentic relationships create vitality and help to grow, even if the therapy is a complex path full of obstacles, partly internal and partly due to family relationships that react to modifications of the existing structures [22]. 

Finally, we suggest that future research should deepen the analysis of the characteristics of social withdrawal from the first years of life, as it can offer valuable information for the clinician who meets the hikikomori child or young adult [45].

## Data Availability

Not applicable.
